# Efficacy of Enamel Matrix Derivative in Vital Pulp Therapy: A Review of Literature

**DOI:** 10.22037/iej.v12i3.12036

**Published:** 2017

**Authors:** Shariq Najeeb, Zohaib Khurshid, Muhammad Sohail Zafar, Sana Zohaib, Fahad Siddiqui

**Affiliations:** a *Restorative Dental Sciences, Al-Farabi Colleges, Saudi Arabia; *; b * School of Metallurgy and Materials, University of Birmingham, Edgbaston, Birmingham, UK; *; c *College of Dentistry, Taibah University, Al Madina Al Munawara, Saudi Arabia; *; d * Department of Biomedical Engineering, College of Engineering, King Faisal University, Al-Hofuf, Saudi Arabia; *; e * Division of Oral Health & Society, McGill College, Montreal, Canada*

**Keywords:** Enamel Matrix Derivative, Pulp Capping, Pulpotomy, Root Canal, Vital Pulp Therapy

## Abstract

**Introduction:**

Vital pulp therapy (VPT) aims to preserve the health and maintain life of the tooth pulp which has been compromised by caries, trauma or restorative procedures. Recently, enamel matrix derivative (EMD) has been introduced as a material for vital pulp therapy. The aim of this review is to critically analyze and summarize the available literature on EMD for VPT.

**Methods and Materials:**

Online databases (PubMED/MEDLINE, Google Scholar, ISI Web of Science, and Wiley-Online) were searched by using the following keywords in various combinations: *Enamel Matrix Derivative, Emdogain, ‘Vital Pulp Therapy, ‘Apexogenisis’, Apexification, Pulp Capping, Endodontics, Dentine *and* Pulpotomy* for studies indexed from January 1949 to April 2016. We used an English-limited search in *Google.co.uk* for the missing grey literature. All studies fulfilling the selection criteria were carefully reviewed for the focused question: “Does using EMD in VPT, compared with other materials, result in better clinical, radiographic and histological outcomes?”.

**Results:**

The primary search resulted in 18 articles of which, 14 articles (including 6 animal studies and 6 clinical trials and 2 case reports) met the inclusion criteria for this review and hence were included. The number of teeth tested in the animal studies ranged from 8 to 144 including pigs, rats and dogs teeth. A number of studies used EMD in the experimental group in comparison with calcium hydroxide, propylene glycol alginate (PGA) and MTA as a control. The observation period ranged from 1 to 2 months and 4 out of 6 animal trials reported more favorable outcomes with EMD while two studies reported comparable outcomes.

**Conclusion:**

Although EMD has potential for various applications in endodontics, studies conducted to date have failed to demonstrate any significant advantage of EMD over conventional VPT materials. Additionally, the 5-year and 10-year survival rate of EMD-treated teeth is not yet known. Hence, studies with a longer follow-up periods are required to deduce the long-term viability of teeth treated with EMD.

## Introduction

Vital pulp therapy (VPT) aims to preserve the life of the healthy tooth pulp which has been compromised by caries, trauma or restorative procedures [1]. Following the removal of carious tooth structure, VPT can be carried out by various means [2]. Apexogenesis and apexification involve the induction of the development of the incomplete root and placement of an artificial barrier or calcifying agent in the open apex of the immature root respectively [3]. Depending on the extent of inflammation, the coronal pulp can be removed to different degrees (from partial to full coronal pulpotomy) [4]. Alternatively, the exposed pulp can be sealed by a biologic capping material, a process known as direct pulp capping (DPC) [5]. Following all these procedures, the tooth is restored with direct (*e.g.* resin composites, dental amalgam, glass ionomer cements, *etc.*) or indirect (*e.g.* porcelain and alloy crowns) restorative materials [2].

Traditionally, calcium hydroxide (CH) has been the material of choice for direct pulp capping (DPC) [6, 7]. CH has the ability to induce the formation of dentine bridge to seal the pulp chamber. Additionally, the alkaline nature of the material is known to have an anti-bacterial and anti-cariogenic effect. However, CH has a number of drawbacks. For instance, the strong alkaline nature has been thought to induce pulpal necrosis, particularly in deciduous teeth [8-10]. Moreover, the efficacy of CH has been challenged by its poor sealing ability, rapid degradation and formation of defects in the underlying dentine bridge [11]. To overcome the aforementioned disadvantages of CH, new materials have been developed. These include mineral trioxide aggregate (MTA), a mixture of tricalcium silicate, tricalcium aluminate and bismuth oxide. Studies indicate that not only MTA induces faster and more marked formation of dentine bridges but it is also clinically easier to use [1, 7]. Nevertheless, weak points such as a prolonged setting time, potential of tooth discoloration and significantly higher cost than CH has limited its use [12]. Pulpotomy of primary teeth also involves the placement of materials such as formocresol and ferric sulphate in addition to MTA and CH following the removal of the coronal pulp [1]. However, due to high toxicity, the use of formocresol has been limited. Ferric sulphate and formocresol can irritate the pulp and are not suitable alternatives to biologic materials used for this purpose [13]. 

More recently, enamel matrix derivative (EMD, Emdogain®) has been advocated for regeneration of dental tissues. EMD is an extract derived from porcine fetal tooth material and mainly consists of amelogenins, a class of proteins known to induce the growth and proliferation of periodontal ligament cells (PDL), along with propylene glycol alginate (PGA) as the degradable carrier [14, 15]. Hence, it has been used as an alloplastic guided tissue regeneration (GTR) material to restore periodontal defects [14, 16-19]. Additionally, animal studies have demonstrated that EMD is more effective than CH in inducing the formation of the dentine bridge and, hence, has the potential to be used in DPC and pulpotomy [20, 21]. Olsson *et al.* [22] have successfully used EMD for DPC material in 8 patients. Nevertheless, Yildirim *et al.* [23] have suggested that EMD has similar clinical efficacy to MTA, CH and formocresol. The aim of this review is to conclude all the studies regarding the efficacy of EMD as a VPT material in animal studies and human clinical trials. Therefore, the focused question for this review is: Does using EMD in VPT, compared with other materials (CH, MTA and formocresol), result in better clinical, radiographic and histological outcomes.

## Materials and Methods

***Search strategy***

The PubMED/MEDLINE, Google Scholar, ISI Web of Science, and Wiley-Online data bases were searched by using the following keywords in various combinations: *Enamel Matrix Derivative, Emdogain, Vital Pulp Therapy, ‘Apexogenesis, Apexification, Pulp Capping, Endodontics, Dentine *and* Pulpotomy,* for studies indexed from 1949 to April 2016. In order to search the missing grey literature, we used *Google.co.uk* and limited the search to English language. Additionally, the relevant articles obtained were read completely for any relevant citations and their reference lists were searched manually for any more pertinent articles.

***Inclusion and exclusion criteria***

The inclusion criteria for the aforementioned search was: Randomized control trials, case series and reports, retrospective studies, *in vivo* animal studies, bibliography of the original and review articles, and studies in English. Likewise the exclusion criteria were: Purely histological (*in vitro*) studies, commentaries, letters to the editor, unpublished articles.

***Appraisal of the literature***

All studies fulfilling the selection criteria were carefully reviewed to find the information related to the focused question; “Does using EMD in VPT, compared with other materials (CH, MTA and formocresol), result in better clinical, radiographic and histological outcomes. Any disagreement among authors was resolved by mutual discussion. The review findings are based on clinical, radiographic and histological outcomes, hence the studies reporting only histological findings were not included in the results.

## Results

The primary search resulted in 18 articles. Of these, 14 articles met the inclusion criteria for this review and hence were included [20-34]. Three studies, not meeting the inclusion criteria, were excluded. Six studies were animal studies [20, 21, 24-27] and eight studies were human trials [22, 23, 28-34]. Two clinical studies were case reports [31, 34]. The included studies have been listed in table 1 and table 2. Studies reporting only histological findings were excluded (Table 3).

All animal studies [20, 21, 24-27] were *in vivo *experiential studies. The number of teeth tested in the animal studies ranged from 8 to 144. Three studies used pigs [20, 21, 27], two studies used rats [25, 26] and one study used dogs [24] as test subjects. Five studies used EMD in the test group [20, 21, 24, 25, 27]. Two studies used CH, one study used propylene glycol alginate (PGA) [25] and one study used MTA as control treatments [27]. One study compared the efficacy of EMD, MTA, platelet-rich plasma (PRP) and CH with that of untreated teeth as negative controls [26]. The observation period ranged from 1 to 2 months [20, 21, 24-27]. Four out of 6 animal studies reported more favorable outcomes with EMD [20, 21, 24, 25] while two studies resulted in comparable outcomes [26, 27]. A number of studies [20, 21, 25] showed dentine formation significantly higher in the experimental group compared to the control. While a few studies [24, 26, 27] showed dentin formation in both groups without any remarkable differences.

In eight human studies [22, 23, 28-34], the number of subjects ranged from 1 to 65 and number of teeth treated with EMD ranged from 1 to 140. The follow-up period ranged from 3 to 24 months and reported clinical and radiographic findings of the treated teeth [22, 23, 28-34]. Two studies [22, 32] assessed clinical, radiographic and histological outcomes after VPT with EMD. Five studies assessed clinical and radiographic outcomes [23, 29-31, 34]. Meanwhile, two studies only evaluated the histological outcomes [28, 33]. Four studies compared the efficacy of EMD for DPC in comparison with CH [22]. In three studies, EMD was used for pulpotomy [23, 28, 29] among which one study did not use any control [28], one used formocresol as control [29] while one study used MTA, formocresol and Portland cement as controls [23]. In one case report, EMD was used in combination with deproteinated bovine bone (Bio-Oss®) for apexification [34]. Olsson *et al. *[22] and Kiatwateeratana *et al. *[32], indicated that EMD was more effective in inducing the formation of the dentine bridge while Fransson *et al. *[33], observed more mineralization when CH was used. All studies resulted in comparable clinical and radiographic outcomes except the one by Olsson *et al.* [22] and Sabbarini *et al.* [29], who had found significantly better outcomes with EMD. These clinical and radiographic findings have been reported on the basis of a very short follow-up period (3 to 24 months). More studies with longer follow period and clinical tails are essential to establish conclusive find about the effect of EMD on pulp tissues. 

## Discussion

The primary constituent of EMD is amelogenin, a group of proteins consisting of ameloblastins, enamelins and tuftelins, all of which have been known to induce tooth formation [38-40]. Additionally, the presence of growth factors such as tissue transforming growth factor beta-1 (TGF-β) in EMD have also been known to stimulate mineralization [41]. Despite the proven *in vitro *effects, EMD has been used for VPT in only a few human and animal studies to date [20-34].

**Table 1:** A summary of animal studies conducted to test the efficacy of enamel matrix derivative placed over exposed pulp

**Study **	**Design**	**Animal **	**N**	**Control treatment**	**Test ** **treatment**	**Observation period**	**Observations and conclusions**
**Nakamura ** ***et al.*** ** [** 20 **]**	*In vivo *	4 pigs	22	CH	EMD	∼1 month	Dentine formation significantly higher in test group.
**Nakamura ** ***et al.*** ** [** 21 **]**	*In vivo *	11 pigs	36	CH	EMD	∼2 months	Dentine formation significantly higher in test group.
**Ishizaki ** ***et al.*** ** [** 24 **]**	*In vivo *	2 dogs	8	No treatment	EMD	∼2 months	Dentine formation observed in test group.
**Igarashi ** ***et al.*** ** [** 25 **]**	*In vivo *	Rats	47	PGA	EMD	∼1 month	Dentine formation significantly higher in test group.
**Orhan ** ***et al.*** ** [** 26 **]**	*In vivo *	36 rats	144	No treatment	EMD, PRP, MTA, CH	∼1 month	Dentine formation comparable in all groups
**Bajić ** ***et al.*** ** [** 27 **]**	*In vivo *	1 pig	20	MTA	EMD	∼1 month	Dentine formation comparable in all groups. No difference in inflammation or presence of bacteria.

*EMD, enamel matrix derivative; Ca(OH)*_2_ *, calcium hydroxide; MTA, mineral trioxide aggregate; PRP, platelet-rich plasma; PGA, propylene glycol alginate*

**Table 2:** ***.*** A summary of clinical studies conducted to test the efficacy of enamel matrix derivative placed over exposed pulp

Study and year	Outcomes assessed	Procedure performed	Subjects (*n*)	Number of teeth (*n*)	Control treatment	Test treatment	Follow-up	Observations and conclusions
Olsson *et al.* [22]	Clinical, radiographic, hisotlogical	Direct pulp capping	8	18 healthy premolars	CH	EMD	∼3 months	EMD enhanced formation of dentine bridge and resulted in lesser post-op symptoms than CH. More inflammation in pulp capped with EMD.
Sabbarini *et al.* [28]	Histological	Pulpotomy	Not stated	10 carious primary canines	No control	EMD	6 months	Dentine bridge formation observed.
Sabbarini *et al.* [29]	Clinical, radiographic	Pulpotomy	15	30 carious primary molars	Formocresol	EMD	6 months	EMD treatment significantly better than formocresol
Garrocho-Rangel *et al.* [30]	Clinical, radiographic	Direct pulp capping	1	1 primary molar	N/A	EMD	12 months	No complications observed at follow up.
Garrocho-Rangel *et al.* [31]	Clinical, radiographic	Direct pulp capping	45	90 carious primary molars	CH	EMD	12 months	2 treatments failed (1 control, 1 test). Control and test outcomes comparable.
Kiatwateeratana *et al.* [32]	Clinical, radiographic, histological	Direct pulp capping	15	30 healthy premolars	CH	EMD	6 months	CH induced more dentine bridge formation and less inflammation. Clinical and radiographic outcomes comparable in both groups.
Fransson *et al.* [33]	Histological	Direct pulp capping	8	18 healthy premolars	CH	EMD	∼3 months	More mineralization observed in control group. More inflammation observed in teeth capped with EMD.
Razavian *et al.* [34]	Clinical, radiographic	Apexification	1	1 carious canine	N/A	EMD+Bio-Oss	24 months	Complete closure of apex. No complications observed at follow-up.
Yildirim *et al.* [23]	Clinical, radiographic	Pulpotomy	65	140 carious primary molars	Formocresol, MTA, Portland cement	EMD	24 months	Clinical outcomes comparable in all groups.

*EMD, enamel matrix derivative; CH, calcium hydroxide; MTA, mineral trioxide aggregate*

**Table 3:** List of studies excluded from this review

Author and year	Reason for exclusion
Lee *et al.* [35]	Only histological study
Min *et al.* [36]	Only histological study
Guven *et al.* [37]	Only histological study

Before biomaterials can be used to treat humans, conducting meticulous *in vitro *and *in vivo *testing are essential [42]. Indeed, as shown in table 1, some animal studies suggest that EMD is more effective than CH [20,21] and MTA [27]. However, the study by Orhan *et al.* [26] has found no difference between the number of osteoblasts and thickness of dentine in teeth capped with EMD, CH, MTA and PRP. Hence, judging the superiority of EMD over other materials in VPT, solely by animal studies, is debatable. It is not possible to carry out long-term animal studies and, hence, none of the animal studies have been observed for more than 2 months [20, 21, 24-27]. Furthermore, all animal studies have used non-carious teeth. Thus, the long-term viability of EMD-treated teeth cannot be deduced from these animal studies. 

Histological evaluation of human capped teeth have not found any conclusive evidence of EMD being superior to other commercially available pulp-capping materials. Olsson *et al.* [22] and Kiatwateeratana *et al.* [32] have found comparable dentine bridge thickness regardless of the capping material. Fransson *et al.* [33] have detected higher levels of expression of dentine sialoprotein (DSP) and collagen I, biomarkers indicative of odontoblast proliferation and dentine formation [43], in human pulp capped with EMD than those capped with CH. On the other hand, a higher level of inflammation of observed with EMD [22, 32, 33] is suggestive of better performance of CH as a pulp capping material. To date, there are no published long-term studies to evaluate the histological effects of EMD on human pulp.

Clinical and radiographic outcomes suggest that the efficacy of EMD is comparable to other pulp capping and pulpotomy materials which include CH, MTA, Portland cement and ferric sulfate [22, 23, 28-34]. Similar conclusions were drawn in a systemic review by Al-Hezaimi *et al.* [44]; however, the review assessed the efficacy of EMD against CH. Although short-term studies indicated that EMD produces lesser post-operative symptoms than CH [22], clinical and radiographic outcomes assessed from 6 to 24 months are comparable regardless of the material used [30-32]. The outcomes of the clinical studies could be the result of the sealing abilities of the restorative materials placed on top of the capping materials. Indeed it has been observed previously that an intact marginal seal is necessary to prevent bacterial leakage and subsequent contamination of the pulp [45]. It has been observed that prompt permanent restoration of teeth following pulp capping, improves the outcomes regardless of the capping material used [43]. Hence, the efficacy of EMD and CH could be due to the sealing effects of the restorative materials rather than the dentine bridge formation. Sabbarini *et al.* [29] have shown that EMD has significantly better results than formocresol after 6 months. However, Yildirim *et al.* [23] have failed to observe any significant advantage of EMD over formocresol, MTA and Portland cement after 2 years of follow-up. These results suggest that although EMD may produce better outcomes after the first few months of placement, its efficacy decreases with time which could be attributed to the inflammatory effects of EMD observed in the aforementioned studies [22, 32, 33]. Indeed, factors such as dentine debris and resin particulates may impede the formation of dentin bridges as they may be a source of bacterial contamination and inflammation [46, 47]. None of the studies reviewed in this review involved pulpal exposures of more than 2 mm [22, 23, 28-34]. Small pulpal exposures would prevent bacterial contamination and may contribute to the success rate of DPC procedures. The gross pulpal exposures are routinely encountered and more deleterious to the pulp. Hence, subsequent clinical trials involving EMD need to involve larger pulpal exposures to be more clinically significant. Razavian *et al. *[34] suggested that EMD and bovine bone (Bio Oss) combination, induced apical closure. Nevertheless, it is not known whether the effects is synergistic or solely due to EMD or Bio Oss. The EMD and Bio Oss are known to promote proliferation of dental pulp cells *in vitro *[48].

In prior studies, PGA, the carrier for EMD, has also been shown to exert an anti-bacterial effect against microorganisms present in dental plaque [49, 50]. Also, although not as high as in teeth capped with EMD, Igarashi *et al.* [25] also observed dentine formation in exposed pulp capped with just PGA. These studies indicate that the efficacy of EMD could be attributed to its carrier as well as amelogenins present in it. The 5 years success rate of DPC is 37% and can be as low as 13% after 10 years [51]. Because the maximum follow-up period recorded in the studies in this review is 24 months, it is difficult to deduce the long-term prognosis of teeth treated with EMD. More studies with longer follow period and clinical tails are essential to establish conclusive find about the effect of EMD on pulp tissues.

## Conclusion

Although EMD has potential in various applications in endodontics, studies conducted to date have failed to demonstrate any significant advantage of EMD over conventional VPT materials. Additionally, the 5-year and 10-year survival rate of EMD-treated teeth is not yet known. Hence, studies with a longer follow-up period are required to deduce the long-term viability of teeth treated with EMD.
